# Sulfonate-Functionalized Mesoporous Silica Nanoparticles as Carriers for Controlled Herbicide Diquat Dibromide Release through Electrostatic Interaction

**DOI:** 10.3390/ijms20061330

**Published:** 2019-03-16

**Authors:** Yongpan Shan, Lidong Cao, Chunli Xu, Pengyue Zhao, Chong Cao, Fengmin Li, Bo Xu, Qiliang Huang

**Affiliations:** 1 Key Laboratory of Integrated Pest Management in Crops, Ministry of Agriculture, Institute of Plant Protection, Chinese Academy of Agricultural Sciences, No. 2 Yuanmingyuan West Road, Haidian District, Beijing 100193, China; shanyongpan@yeah.net (Y.S.); springxcl2013@126.com (C.X.); pengyue_8825@163.com (P.Z.); ccao@ippcaas.cn (C.C.); fmli@ippcaas.cn (F.L.); 2 Henan Provincial Engineering and Technology Research Center for Controlled Pesticide and Fertilizer Release, Henan Haonianjing Biological Development Co., Ltd., Yangjin Industrial park, Jinshui District, Zhengzhou 450000, China; haonianjing01@163.com

**Keywords:** mesoporous silica nanoparticles, diquat dibromide, electrostatic interaction, controlled release, bioactivity

## Abstract

Environmental stimuli-responsive pesticide release is desirable for enhanced efficiency and reduced side effects. In most cases, the loading and release of pesticides mainly depends on hydrophobic interactions and hydrogen bonding. Electrostatic interaction is less investigated as a weapon for achieving high loading content and controlled pesticide release. In this work, negative-charge decorated mesoporous silica nanoparticles (MSNs) were facilely fabricated by introducing sulfonate groups onto MSNs through a post-grafting method. Sulfonate-functionalized MSNs (MSN-SO_3_) were synthesized by conversion of epoxy group into sulfonate group using a bisulfite ion as a ring opening reagent. Diquat dibromide (DQ), one of the globally used quaternary ammonium herbicides, was efficiently loaded into these negatively charged MSN-SO_3_ nanoparticles. The loading content was increased to 12.73% compared to those using bare MSNs as carriers (5.31%). The release of DQ from DQ@MSN-SO_3_ nanoparticles was pH and ionic strength responsive, which was chiefly governed by the electrostatic interactions. Moreover, DQ@MSN-SO_3_ nanoparticles exhibited good herbicidal activity for the control of *Datura stramonium* L., and the bioactivity was affected by the ionic strength of the release medium. The strategy of cargo loading and release dependent on the electrostatic interactions could be generally used for charge-carrying pesticides using carriers possessing opposite charges to mitigate the potential negative impacts on the environment.

## 1. Introduction

**Figure 1:**
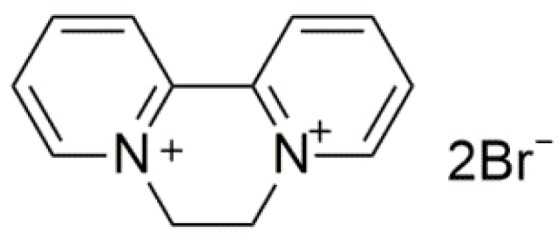
Chemical structures of diquat dibromide (DQ).

Pesticides play important roles in the high food productivity and farmers’ income through the control of biological disasters. Herbicides are chemicals applied to eliminate losses of weed interference on crops, leading to substantial agronomic and economic benefits [1,2]. Diquat dibromide (DQ, Figure 1), which belongs to the category of bipyridines, is globally used as a cationic quaternary ammonium herbicide [3]. DQ is widely applied for the control of aquatic weeds due to its low cost and high efficiency [4]. However, the massive agricultural use of DQ not only increases the cost, but also results in negative impacts on the environment [5,6]. To address this limitation, the exploitation of the controlled release formulations (CRFs) of DQ could be promising, since CRFs permit the application of minimal amounts of an active ingredient to maintain the desirable activity, which will mitigate the potentially negative impacts on the environment, and promote sustainable agricultural development [7,8,9,10].

From a macro-level perspective, the release profile of cargo molecule is closely related to the material used as a carrier [11,12]. Recently, various biodegradable materials including natural poly- and oligosaccharides [13,14], polyhydroxyalkanoates [15], biochar [16], clays and double hydroxides [17,18], are widely used as carriers for plant protection compounds. Since the discovery of Mobil Crystalline Material 41 [19], considerable research attention has been paid on mesoporous silica nanoparticles (MSNs) due to MSNs’s remarkable characteristics—such as low cost, facile preparation, biocompatibility, controllable mesoporous and surface structure, and high hydrothermal stability [20,21]. Taking advantage of these remarkable characteristics, MSNs as ideal scaffoldings for delivery systems have been studied extensively in the pharmaceutical industries for the release of active substances [22,23,24]. Recently, MSNs have also been extensively used as carriers to control the release of pesticides in the agriculture field [25,26,27,28,29,30]. During the pursuit of an ideal carrier for a pesticide, our group has also prepared surface-decorated versatile MSNs and investigated the controlled release profiles of cargo molecules [31,32,33]. Moreover, we also studied the translocation, distribution and degradation patterns of pesticide encapsulated MSNs in cucumber plants [34,35].

From a micro-level perspective, the release profile of a cargo molecule is mainly governed by the noncovalent (e.g., hydrophobic, hydrogen bonding, and ionic) interaction between the carrier material and cargo molecule [36]. Although MSNs as carriers to control pesticide release have made some progress, the loading and release of pesticides mainly depend on the hydrophobic interaction and hydrogen bonding. Ionic interactions are the interactions involving electrostatic repulsion and attraction between oppositely charged ions, which are supposed to be stronger than hydrophobic interactions and hydrogen bonding [37]. They are less studied as a strategy for obtaining, and approving loading content and controlled release of pesticides. For charge carrying pesticides, the loading content and release pattern should be increased and regulated through the electrostatic interaction by surface modification of the MSNs to carry more opposite charges. 

Recently, we have prepared positive-charge functionalized MSNs by introducing trimethylammonium groups onto MSNs by a post-grafting method [38]. Making most of the ionic interactions, herbicide of 2, 4-dichlorophenoxy acetic acid (2, 4-D) was loaded with high content. More importantly, pH, temperature, and ionic strength-responsive release profiles were achieved, which were mainly governed by the electrostatic interactions. Consequently, for positive charge carrying herbicide DQ, modification of MSNs with negative charge is expected to increase the loading content by enhancing electrostatic attraction. 

**Figure 2:**
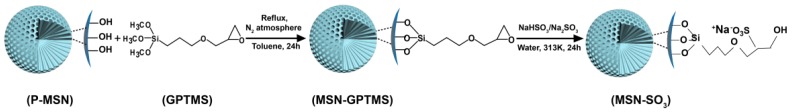
Schematic illustration of the synthetic route of MSN-SO_3_ nanoparticles.

In this work, MSNs were modified with sulfonate groups via conversion of epoxy group into sulfonate group using a bisulfite ion as a ring opening reagent, as seen in Figure 2. The as-prepared negative-charge functionalized MSNs (MSN-SO_3_) were used as carrier materials for loading DQ, and the loading content is greater than unmodified pristine MSNs (P-MSN). The parameters that effect the loading content such as the solvent and ratio of carrier to pesticide were studied. In addition, the release profiles were also studied under different conditions including ionic strength and pH value. Finally, the herbicidal activity of DQ-loaded MSN-SO_3_ (DQ@ MSN-SO_3_) against target plant datura (*Datura stramonium* L.) was investigated.

## 2. Results and Discussion

### 2.1. Preparation and Characterization of Nanoparticles

**Figure 3:**
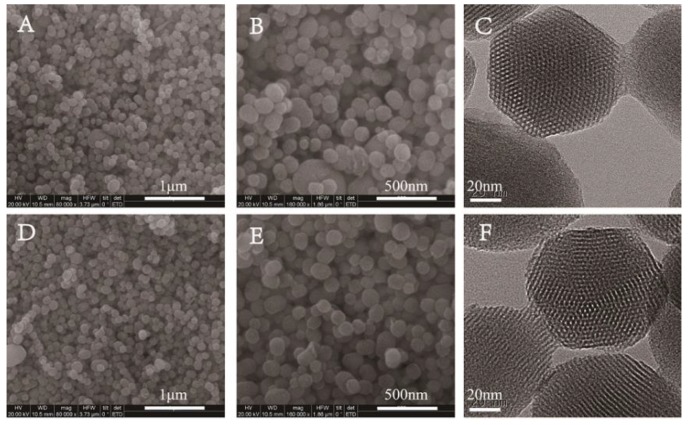
Scanning electron microscopy (SEM) (**A**,**B**,**D**,**E**) and transmission electron microscopy (TEM) (**C**,**F**) images of nanoparticles. Pristine MSN (P-MSN) (**A**–**C**); sulfonate-functionalized MSNs (MSN-SO_3_) (**D**–**F**). Scale bars: (**A**,**D**) 1.0 μm, (**B**,**E**) 500 nm, and (**C**,**F**) 20 nm.

P-MSN was prepared with a liquid crystal templating method under basic reaction conditions using tetraethyl orthosilicate (TEOS), as the silica source and cetyltrimethylammonium bromide (CTAB) as the structure-directing agent. The organic groups on the surfaces of MSN-GPTMS were introduced by post-grafting strategy using 3-glycidoxypropyltrimethoxysilane (GPTMS) as the silane agent. Subsequently, MSN-SO_3_ nanoparticles were synthesized via conversion of epoxy group into the sulfonate group using a bisulfite ion as a ring-opening reagent. The schematic illustration of the synthetic routes of MSN-SO_3_ nanoparticles is shown in Figure 2. The shape and size of P-MSN and MSN-SO_3_ nanoparticles were determined by scanning electron microscopy (SEM) and transmission electron microscopy (TEM) observations (Figure 3), which showed that the nanoparticles were to be globular in morphology with a smooth surface, and there were no significant morphological differences between P-MSN (Figure 3A,B) and MSN-SO_3_ (Figure 3D,E). In addition, the binding of organic groups onto the surface of P-MSN did not obviously change the particle size, with the average diameters of 142 and 148 nm, respectively, estimated by the statistical analysis of the SEM images of 200 randomly selected samples. Moreover, the TEM micrographs (Figure 2C,F) showed that the well-ordered mesoporous structures of the nanoparticles did not show obvious changes before and after modification. These results indicated that the organic groups can be easily incorporated on P-MSN by the grafting method. 

**Figure 4:**
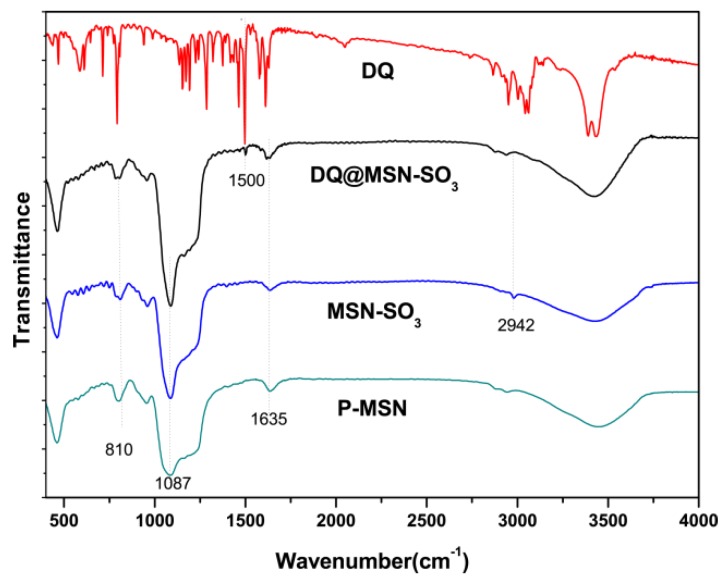
Fourier transform infrared (FT-IR) spectra of P-MSN, MSN-SO_3_, DQ, and DQ@MSN-SO_3_.

Fourier transform infrared (FT-IR) spectra were used to study the organic side groups present in the framework of P-MSN after modification. As shown in Figure 4, the peaks at 810 and 1087 cm^−1^ in P-MSN, MSN-SO_3_ and DQ@MSN-SO_3_ could be attributed to the stretching vibration of Si–O–Si bonds of the condensed silica network. The absorption band at 2942 cm^−1^ can be assigned to C–H stretching vibrations in the alkyl chain, which confirmed that the organic groups were indeed incorporated on the surface of P-MSN by covalent bonding. However, the absorption band of asymmetric and symmetric stretching vibrations of sulfonate group could not be confirmed due to their overlap with stretching vibration of Si–O–Si. bonding. 

**Figure 5:**
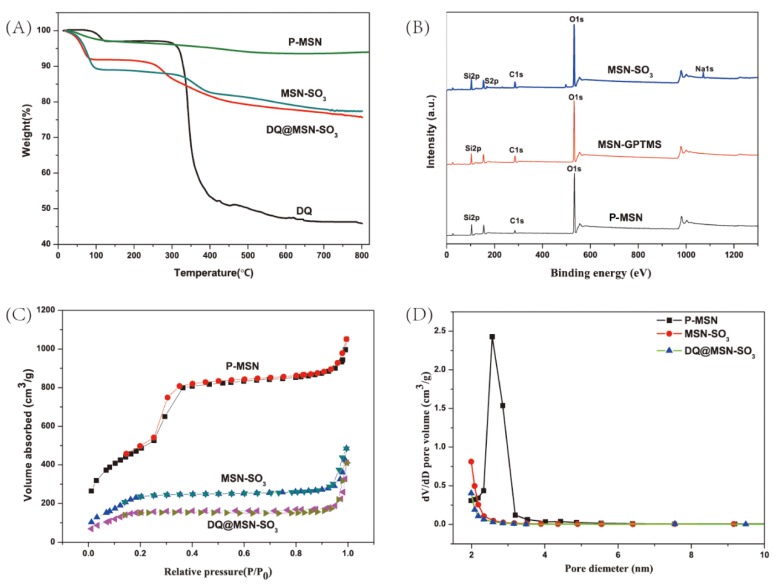
Thermogravimetric analysis (TGA) curves (**A**), X-ray photoelectron spectroscopy (XPS) spectra (**B**), Nitrogen adsorption-desorption isotherms (**C**), and pore size distributions (**D**) of nanoparticles.

Thermogravimetric analysis (TGA) is a method to measure the relationship between samples weight and temperature under program control temperature. It is often used to investigate the thermal stability and thermal decomposition behaviors of materials. In this work, TGA was performed for P-MSN, MSN-SO_3_, DQ, and DQ@MSN-SO_3_ (Figure 5A). All samples show different weight loss at temperatures below 100 °C, which could be considered the loss of water absorbed by the samples. In the temperature range from 100 to 800 °C, the weight of P-MSN is reduced by about 4%, and this may be due to the decomposition of residual CTAB in the samples. About 11% of the weight loss of MSN-SO_3_ at temperatures ranging from 300 to 800 °C is attributed to the decomposition of organic groups on the surface of MSN, which further confirms that P-MSN was successfully functionalized with organic groups. In the same temperature range, the weight loss of DQ@MSN-SO_3_ is about 6% more than MSN-SO_3_, and this is mainly due to the decomposition of DQ loaded on MSN-SO_3_.

X-ray photoelectron spectroscopy (XPS) is commonly used to determine the elements on the surface of the material. It can provide accurate information about the elements on the surface of nanoparticles. As shown in Figure 5B, the peaks of O1s, C1s and Si2p appear in the XPS spectrum of P-MSN, the binding energies are approximately 532.8, 284.8 and 103.5 eV, respectively. The weaker signal at 284.8 eV could be derived from the carbon residue of the template after calcining. Especially, in the XPS curve of MSN-SO_3_, the peaks at 168.2 and 1072.1 eV correspond to the S2p and Na1s, which confirmed that the sulfonate group has been successfully incorporated into the surface of MSN nanoparticles. The atomic percentage of S and Na were 2.35% and 2.33%, respectively.

The N_2_ adsorption–desorption isotherms and pore size distribution curves for P-MSN, MSN-SO_3_, and DQ@ MSN-SO_3_ are shown in Figure 5C,D. The values for Brunauer–Emmett–Teller (BET) specific surface area (S_BET_), total pore volume (V_t_), and Barrett-Joyner-Halenda (BJH) pore diameter (D_BJH_) of the materials are summarized in Table 1. The type IV isotherm curve with a hysteresis loop between 0.3 and 0.4 of P/P0, and the pore size distribution plot displaying a narrow distribution at 2–3 nm indicate the existence of well-ordered mesoporous structures of P-MSN [39]. Compared with unmodified MSN, functionalization of MSN with organic groups led to the decrease of specific surface area from 1767.5 to 973.1 m^2^/g, and the pore volume decreased from 1.4 to 0.5 cm^3^/g, which is possibly due to the partial occupation of the pores by the organic groups [40]. When DQ was loaded into MSN-SO_3_, the specific surface area (from 973.1 to 623.3 m^2^/g) and the total pore volume (from 0.5 to 0.3 cm^3^/g) were further reduced compared to MSN-SO_3_, which indicates that DQ was successfully loaded into nanoparticles and less space was left for nitrogen adsorption [39].

Zeta potential measurements were used to detect the surface charge of the prepared samples. The zeta potential of P-MSN, MSN-SO_3_ and DQ@MSN-SO_3_ samples were measured in aqueous solution. The unmodified P-MSN carried a negatively charged surface with a zeta potential of −20.2 mV, which could be due to the deprotonation of the Si-OH groups on the surface of the P-MSN. The zeta potential of MSN-SO_3_ was further decreased to −37.0 mV due to the introduction of abundant negatively charged sulfonate group. As expected, the zeta potential of DQ@MSN-SO_3_ was increased to −17.9 mV due to the loading of positively charged DQ. In addition, the particle size of the nanoparticles were also measured based on dynamic light scattering, and the average diameters of P-MSN, MSN-SO_3_, and DQ@MSN-SO_3_ were 415.3, 267.5 and 240.8 nm, respectively, which were higher than that measured by SEM observation (Table 1). This phenomenon is possibly due to the hydrate of nanoparticles or aggregates in solution. 

### 2.2. Loading of DQ into MSN-SO_3_ Nanoparticles

When negatively charged MSN-SO_3_ were in hand, the loading content (LC) and encapsulation efficiency (EE) of herbicide DQ were then optimized under different conditions including the solvent and the ratio of carrier to pesticide. The LC and EE results under screened conditions are summarized in Table 2. Due to the good solubility of DQ in H_2_O, H_2_O was firstly adopted as a solvent to test the loading experiment. When P-MSN were used as carriers, the LC was 5.31% (entry 1, Table 2). As expected, the LC increased obviously to 12.73% when more negatively charged MSN-SO_3_ were used (entry 2, Table 2). These results clearly indicate that electrostatic attraction plays an important role in pesticide loading. When 80% aqueous ethanol or 80% aqueous acetonitrile was used as the solvent, the LC was 14.69% and 13.81%, respectively (entry 3 and 4, Table 2). There was no significant change compared with that using water as the solvent. Generally, a higher concentration of a cargo molecule in solution can generate a stronger gradient to facilitate its diffusion into the MSN pores [36]. However, when the dosage of DQ doubled, the LC only increased from 12.73% to 13.98% with sacrificing the EE from 13.48% to 6.99% (entries 2 and 5, Table 2), which indicated a saturation absorption of DQ. Taking the LC, EE, and cost into comprehensive consideration, the conditions of pure water as a solvent and the DQ/carrier ratio of 1:1 were used for the scale preparation of DQ@MSN-SO_3_, which were applied for the release study and herbicidal activity assay. 

### 2.3. Controlled Release of DQ 

**Figure 6:**
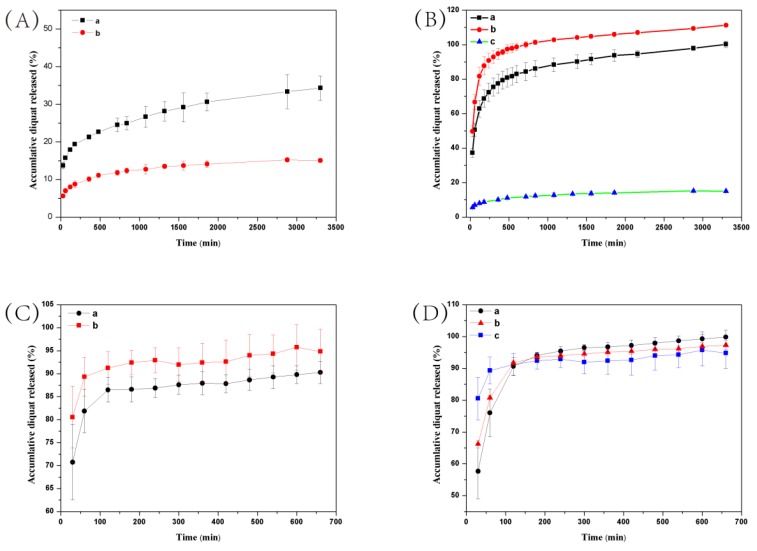
Release profiles of DQ from DQ@MSN-SO_3_ (**A**,**B**) and DQ aqueous solution (**C**,**D**) at different pH values (**A** and **C**, a: pH = 3, b: pH = 7) and ionic strengths (**B** and **D**, a: 0.1 M Na_2_SO_3_, b: 0.2 M Na_2_SO_3_, c: water). Error bars correspond to standard errors of triplicate measurements.

**Figure 7:**
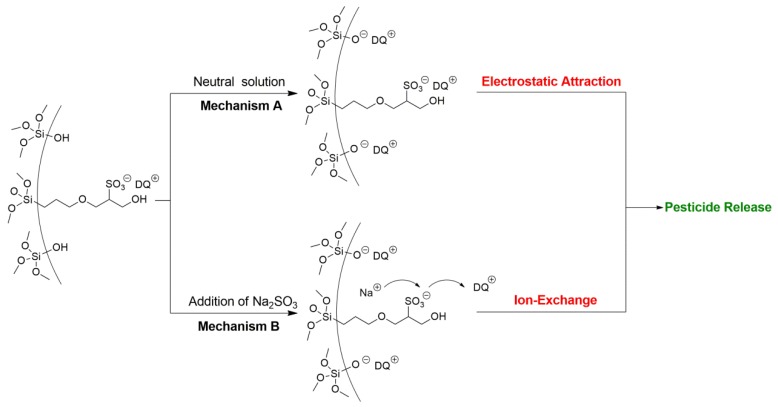
Schematic illustration of the controlled-release mechanism of a cationic herbicide DQ loaded in negative-charge functionalized MSNs. Mechanism A: pesticide released by electrostatic interaction under different pH solution. Mechanism B: Pesticide released through ion-exchange by increasing the ionic strength.

Environmental stimuli-responsive pesticide release is desirable for enhanced efficiency and reduced side effects. After successful preparation of DQ@MSN-SO_3_ samples, the release profiles of DQ under different conditions including pH and ionic strength was investigated. In this study, the effect of pH on release performance was studied by adjusting the pH values of the release medium with hydrochloric acid or sodium hydroxide. As shown in Figure 6A, the release behavior of DQ was obviously pH-sensitive. The accumulative release of DQ from DQ@MSN-SO_3_ reached 47% after 55 h under pH 3. However, the corresponding amount of release was merely 20% under pH 7. This result showed that the release was quicker in an acidic medium than that in a neutral environment. It is clearly indicated that the electrostatic interactions governed the release behaviors with the changing of the environmental pH. The schematic illustration of the controlled-release mechanism of a cationic herbicide DQ is shown in Figure 7. In a neutral solution, the MSN-SO_3_ nanoparticles possess more negative charge, and a strong electrostatic attraction retarded the release of positively charged DQ (mechanism A, Figure 7). In an acidic solution, the negatively charged sulfonate and SiO^-^ groups were protonated. Consequently, electrostatic repulsion became dominant and the release of DQ was accelerated. This pH-responsive release pattern can be beneficial in acidic soil environment which facilitates the release of DQ. 

The effect of ionic strength of the solution on the release behavior of DQ was also investigated. Aqueous Na_2_SO_3_ solution and water as the control were adopted as the release medium to study the effect of ionic strength on the release performance. As shown in Figure 6B, the accumulative release of DQ was only 10% after 6 h in pure water. However, the corresponding amount of release reached 77% in 0.1M Na_2_SO_3_ aqueous solution at the same time interval. When the concentration of Na_2_SO_3_ aqueous solution improved to 0.2 M, the amount of release increased to 94%, which clearly demonstrated the ionic strength-dependent release pattern. This ionic strength-triggering release is primarily attributed to the ion-exchange mechanism (mechanism B, Figure 7). With the increasing amount of Na^+^ in the release medium, the competitive electrostatic attraction between negatively charged sulfonate and SiO^−^ groups and positively charged Na^+^ would compel DQ to be far away from the surface of nanoparticles, thus facilitating the release of DQ. When commercially available DQ aqueous solution was tested, the release was faster than that of DQ@MSN-SO_3_. Moreover, the release of DQ did not show obvious dual pH and ionic strength responsive characteristics (Figure 6C,D).

Barrett reported the preparation of insoluble gel of calcium alginate from sodium alginate and calcium ions [41]. DQ could be loaded into the alginate gel and be slowly released into the water close to the plants. Liu et al. fabricated carboxymethyl-β-cyclodextrin functionalized magnetic adsorbents, which could regulate the DQ release by changing the pH values [42]. The dual pH and ionic strength responsive release developed in the present study can find potential applications of such a herbicide in sustainable plant protection. 

### 2.4. Bioassay of DQ@MSN-SO_3_ nanoparticles.

**Figure 8:**
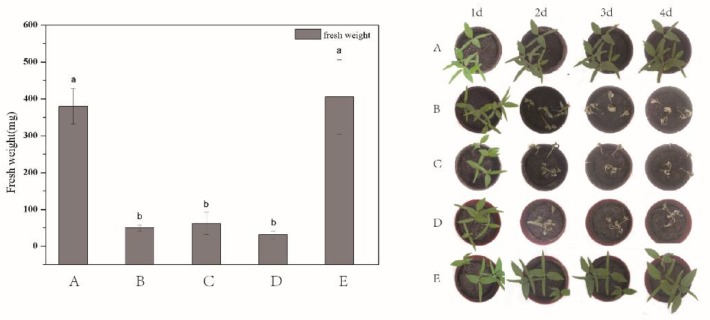
Herbicidal activity for target plant datura (*Datura stramonium* L.) determined in terms of fresh weight: (A) water, (B) free DQ technical in water, (C) DQ@MSN-SO_3_ in water, (D) DQ@MSN-SO_3_ in 0.1M Na_2_SO_3_ aqueous solution, and (E) 0.1M Na_2_SO_3_ aqueous solution. Error bars correspond to standard errors of triplicate measurements. Bars marked with different letters are statistically different at *p* ≤ 0.05 as determined by Duncan’s multiple range test.

In order to test the applicability of the as-prepared DQ@MSN-SO_3_ nanoparticles, the herbicidal activity against target plant datura (*Datura stramonium* L.) in terms of fresh weight was evaluated. To better throw light on the subject, the bioactivity of water (Figure 8A) and 0.1 M Na_2_SO_3_ aqueous solution (Figure 8E) as negative controls, free DQ technical in water (Figure 8B) as positive control, and DQ@MSN-SO_3_ nanoparticles in different solvents including pure water (Figure 8C) and 0.1 M Na_2_SO_3_ solution (Figure 8D) were compared. As shown in the histogram in Figure 8, the herbicidal activity of DQ@MSN-SO_3_ nanoparticles in water was slightly lower than that of free DQ technical, and the fresh weight was reduced 83% compared to the treatment with water after five days of application. However, the herbicidal activity of DQ@MSN-SO_3_ was enhanced when 0.1M Na_2_SO_3_ aqueous solution was used as a solvent, and the fresh weight was reduced to 91% compared to the treatment with water. These results demonstrated that DQ@MSN-SO_3_ nanoparticles had good herbicidal activity and was affected by the ionic strength of the release medium. The bioactivity results indirectly confirmed the ionic strength-triggering release characteristics of DQ@MSN-SO_3_ nanoparticles.

## 3. Materials and Methods 

### 3.1. Materials

Sodium sulfite (Na_2_SO_3_, 97%), sodium bisulfite (NaHSO_3_, 99%), and cetyltrimethylammonium bromide (CTAB, 99%) were obtained from Sinopharm Chemical Reagent Co, Ltd. (Beijing, China). Diquat dibromide (DQ, 41.5%) was purchased from Shandong LvFeng Pesticide Co. Ltd. (Qingzhou, Shandong province, China). 20% DQ aqueous solution was obtained from Shandong Luba Chemical Co., Ltd. (Jinan, China). Tetraethyl orthosilicate (TEOS) with the purity of 99% was obtained from Fluorochem Ltd. (Hadfield, UK). 3-Glycidoxypropyltrimethoxysilane (GPTMS, 97%) was purchased from J&K Scientific Ltd. (Beijing, China). All other chemicals were commercially available and used as received. Deionized water used for reactions and the treatment process were attained from a Milli-Q water system (Millipore Corporation, Bedford, MA, USA). 

### 3.2. Synthesis of the Nanoparticles

#### 3.2.1. Synthesis of Pristine Mesoporous Silica Nanoparticles (P-MSN) 

The synthesis of P-MSN followed a typical sol-gel method reported by Radu et al. with a slight modification [43]. Briefly, 2.0 g of CTAB was dissolved in 960 mL of deionized water in a three-neck flask, and then 7 mL of 2.0 M NaOH was added slowly at room temperature under stirring at 800 rpm. The reaction mixture was heated to 80 °C in an oil bath, and then 10 mL of TEOS was added dropwise. Afterwards, the mixture was continuously stirred at 80 °C for 6 h. The white solid precipitate produced during the reaction was filtered, collected, and washed three times with ethanol and water, respectively. The washed sample was dried overnight in an oven at 80 °C. In order to remove unreacted CTAB, the dried sample was calcined at 550 °C for 5 h.

#### 3.2.2. Synthesis of Sulfonate-Functionalized MSN (MSN- SO_3_)

Sulfonate-functionalized MSNs (MSN-SO_3_) were prepared according to the method reported by Badiei with little modification [44]. Firstly, the surface of P-MSN was functionalized by 3-glycidoxypropyltrimethoxysilane (GPTMS) using a post-grafting synthesis. Typically, 1.0 g of P-MSN was suspended in 50 mL of anhydrous toluene, and then 1 mL of GPTMS was added under the protection of nitrogen. The resulting mixture was refluxed at 120 °C for 24 h. Then, the white solid was separated by centrifugation at 8000 rpm for 10 min and washed three times with toluene and ethanol, respectively, to remove the unreacted materials. The as-prepared samples (MSN-GPTMS) were dried overnight in an oven at 60 °C.

Subsequently, MSN-SO_3_ were synthesized via conversion of epoxy group into sulfonate group using bisulfite ion as ring opening reagent. Briefly, 1.0 g of MSN-GPTMS was dissolved in 30 mL 3 M aqueous solution of Na_2_SO_3_ and NaHSO_3_ (NaHSO_3_:Na_2_SO_3_ = 3:2 ratio), the mixture was stirred at 40 °C for 12 h. The resultant samples (MSN-SO_3_) were filtered and washed three times with water to remove unreacted sulfite ions. Then the product was dried overnight in an oven at 60 °C.

### 3.3. Sample Characterization 

Fourier transform infrared (FT-IR) spectra of the samples were performed on an infrared spectrophotometer (NICOLET 6700, Thermo Fisher Scientific, Waltham, MA, USA) using potassium bromide pellets in the spectral region of 4000 to 400 cm^−1^. Thermogravimetric analysis was conducted using a thermogravimetric analyser (Perkin Elmer Pyris Diamond, Woodland, CA, USA) under N_2_ atmosphere at a heating rate of 20 °C/min from 30 to 800 °C.

The specific surface area and pore size distribution of the nanoparticles were explored by measuring the nitrogen adsorption with a specific surface area analyzer (TriStarII 3020, Micromeritics Instruments Corp, Norcross, GA, USA) at −196 °C. Prior to measurement, the samples were degassed at 10^−3^ Torr and 120 °C for 6 h. The Brunauer–Emmett–Teller (BET) equation was used to calculate the specific surface area from the linear section of the BET curve, and the pore size distribution was assessed with the Barrett-Joyner-Halenda (BJH) model from the adsorption branches of the isotherm.

The morphological features and particle size of the as-synthesized nanoparticles were observed by scanning electron microscopy (SEM, SU8000, Hitachi Ltd., Tokyo, Japan) at 10 kV and transmission electron microscopy (TEM, Tecnai G2, F20 S-TWIN, FEI, Oregon, USA) at 200 kV. The chemical elemental compositions of the samples were determined using an X-ray photoelectron spectrometer (ESCALab 250Xi, Thermo Fisher Scientific, USA) using 150 W monochromatic Al Kα radiation (1486.6 eV, 500 µm spot size) as the excitation source. All of the other binding energies were calibrated by the C1s peak at 284.8 eV

The zeta potential and particle size of nanoparticles were determined on a ZetaSizer Nano ZS Analyzer (Malvern Instruments Ltd., Malvern, UK) based on dynamic light scattering. The sample solutions of 1 mg/mL were prepared using distilled water as solvent to make concentrations in the range suitable for scattering. Prior to measurement, the sample solutions were ultrasonicated for 5 min to make it evenly dispersed.

### 3.4. Loading of DQ into MSN-SO_3_

DQ was loaded into MSN-SO_3_ according to literature with a little modification [45]. Generally, 30 mg of MSN-SO_3_ were dispersed in 5 mL of aqueous solution of DQ at the concentration of 6.0 mg/mL. The suspension was stirred at 700 rpm for 6 h at room temperature, and the supernatant was then removed by centrifugation at 8000 rpm for 10 min. The resulting precipitates were washed one time with 1 mL of distilled water to remove unloaded DQ The DQ-loaded MSN-SO_3_ nanoparticles were denoted as DQ@MSN-SO_3_, which were dried overnight in an oven at 60 °C. The loading content of DQ was determined by a direct method. Briefly, about 20 mg of DQ@MSN-SO_3_ were weighed and washed with 5 mL of 0.1 M aqueous solution of Na_2_SO_3_ by sonification for 30 min at room temperature, and supernatant was then collected to by centrifugation at 8000 rpm for 5 min, and this treatment was repeated three times. 

The concentration of DQ in the combined supernatant was determined on high-performance liquid chromatography (HPLC, 1200-DAD (diode array detector), Agilent Technologies, Santa Clara, CA, USA). The loading content LC (%) of DQ was calculated as follows: Loading content (%) = (weight of DQ in DQ@MSN-SO_3_/weight of DQ@MSN-SO_3_) ×100%. The operating parameters for HPLC analysis were as follows: SB-C18 reversed-phase column (5 μm × 4.6 mm × 150 mm); column temperature, 30 °C; mobile phase, (acetonitrile/20 mmol aqueous solution of potassium hexafluorophosphate and triethylamineaqueous (V/V) = 5:95, adjust pH to 3 with phosphoric acid); flow rate, 1.0 mL/min; and DAD signals at 307 nm.

### 3.5. In Vitro Release of DQ

The release behavior of DQ was investigated on a dissolution tester (D-800LS, Tianjin University, Tianjin, China). Generally, about 20 mg of DQ@MSN-SO_3_ samples were dispersed in 3 mL of release medium in dialysis bags (molecular weight cutoff, 8000–14,000) at 30 °C. The sealed dialysis bag was immersed in 197 mL of release medium at a stirring speed of 100 rpm. At given time intervals, 1 mL of the release medium was taken out for HPLC analysis. Meanwhile, 1 mL of fresh release medium was supplemented to make a constant total solution volume. To investigate the influence of pH values on the release profiles, three different pH values of 3, 5, and 7 of release medium were adjusted using diluted HCl and NaOH. For the effect of ionic concentration on the release profiles to be investigated, the different concentrations of Na_2_SO_3_ aqueous solutions (0, 0.1 and 0.2 M) were used as release medium. Experiments under different conditions were repeated three times, respectively. The accumulative DQ released was calculated with the following formula:

(1) $$E_{r}=\frac{V_{e}\sum_{i=0}^{n-1}C_{i}+V_{0}C_{n}}{m_{pesticide}}\times 100\%$$

where *E_r_* is the accumulative release (%) of DQ with regard to the total DQ loaded; *V_e_* is the volume of the release medium withdrawn at a predetermined time (*V_e_* = 1 mL); *V*_0_ is the total volume of release medium (*V*_0_ = 200 mL); *C_n_* (mg/mL) is the concentration of DQ in release medium at time n; *m_pesticide_* (mg) is the total amount of DQ loaded in DQ@MSN-SO_3_.

### 3.6. Bioactivity Studies of DQ@MSN-SO_3_

The herbicidal activity of the DQ-loaded nanoparticles was determined using a laboratory bioassay against *Datura stramonium* L. according to our previous reported method [38]. For the test, ten seeds of *Datura stramonium* L. were sown in each pot (10 cm high with a diameter of 9.0 cm) which was filled with about 250 g of nutrient soil and grown in the greenhouse. In order to maintain moisture, the pots were watered daily with the same volume of water. Subsequently, the *Datura stramonium* L. seedlings that did not grow well were pulled up. However, no less than three plants per pot were guaranteed. When the plant had four true leaves, the solution of DQ@MSN-SO_3_ samples was applied with a dose equal to the field application (1.1 kg/ha for DQ). The applied active ingredient of DQ for each pot was 0.9 mg. Equal dose of free DQ technical and treatment without herbicide as positive and negative controls were used. Each treatment was performed triplicate. Five days after the application of herbicide, the fresh weights of the aerial part of the Datura stramonium L. seedlings were measured to assess the bioactivity DQ@MSN-SO_3_ samples.

### 3.7. Statistical Analysis

Statistical analysis was performed with SPSS 10.0 software (SPSS Inc., Chicago, IL, USA). One way analysis of variance (ANOVA) followed by Duncan’s multiple range test (DMRT) were used. The values are mean ± standard deviation (SD) for triplicate determinations in each group. P values less than or equal to 5% were considered as significant. 

## 4. Conclusions

In conclusion, a stimuli-responsive cationic herbicide DQ release system has been designed by introducing negative charges on the surface of MSN. Surface functionalization was confirmed by FT-IR, TGA, XPS, N_2_ adsorption desorption, and zeta potential analyses. Electrostatic attractions are the driving forces facilitating DQ loading. The release of DQ from DQ@MSN-SO_3_ was pH and ionic strength responsive. The release was quicker in the acidic medium than that in neutral environment. Improving the ionic strength of the release medium led to faster DQ release mainly due to the ion-exchange mechanism. Moreover, DQ@MSN-SO_3_ nanoparticles exhibited good herbicidal activity for the control of *Datura stramonium* L., and the bioactivity was affected by the ionic strength of the release medium. The cargo loading and release based on the strategy of electrostatic interactions could be generally applied to charge-carrying plant protection compounds using carriers possessing opposite charges to mitigate the potential side effects on the environment.

## Figures and Tables

**Table 1:** Mesoporous structure characterization of nanoparticles ^a^.

Sample	S_BET_ (m^2^/g)	V_t_ (cm^3^/g)	D_BJH_ (nm)	Size (nm)	PDI	Zeta (mV)
P-MSN	1767.5	1.4	3.3	415.3 ± 148.08	0.56 ± 0.23	−20.2 ± 0.38
MSN-SO_3_	973.1	0.5	2.1	267.5 ± 7.15	0.28 ± 0.03	−37.0 ± 0.40
DQ@MSN-SO_3_	623.3	0.3	/	240.8 ± 5.96	0.22 ± 0.04	−17.9 ± 0.15

^a^ SBET, Brunauer–Emmett–Teller (BET) specific surface area; V_t_, total pore volume; D_BJH_, BJH pore diameter; PDI, polydispersity index.

**Table 2:** Loading content (LC) and encapsulation efficiency (EE) of DQ into MSN-SO_3_ nanoparticles ^a^.

Entry	Carrier	Solvent	Mass Ratiob	LC (%)	EE (%)
1	P-MSN	H_2_O	1.0	5.31 ± 0.12	5.14 ± 0.09
2	MSN-SO_3_	H_2_O	1.0	12.73 ± 0.02	13.48 ± 0.02
3	MSN-SO_3_	80% aqueous EtOH	1.0	14.69 ± 0.10	15.99 ± 0.17
4	MSN-SO_3_	80% aqueous MeCN	1.0	13.81 ± 0.04	16.01 ± 0.08
5	MSN-SO_3_	H_2_O	2.0	13.98 ± 0.11	6.99 ± 0.05

^a^ Values are mean ± SD of three replicates. ^b^ Mass ratio of DQ to carrier.
